# Capturing pharmacists’ impact in general practice: an e-Delphi study to attempt to reach consensus amongst experts about what activities to record

**DOI:** 10.1186/s12875-019-1008-6

**Published:** 2019-09-09

**Authors:** Georgios Dimitrios Karampatakis, Kath Ryan, Nilesh Patel, Graham Stretch

**Affiliations:** 1 School of Pharmacy, University of Reading, Whiteknights Campus, PO Box 226, Reading, RG6 6AP UK; 2 Ealing GP Federation, 179C Bilton Road, Perivale, Greenford, Middlesex, UB6 7HQ UK

**Keywords:** Pharmacist, General practice, UK, Key performance indicators, Delphi study, Activity codes

## Abstract

**Background:**

In the UK, there is ongoing integration of pharmacists into general practice as a new healthcare service in primary care. Evaluation of the service involves national measures that require pharmacists to record their work, on the general practice clinical computer systems, using electronic activity codes. No national agreement, however, has been established on what activities to record. The purpose of this study was to attempt to reach consensus on what activities general practice-based pharmacists should record.

**Methods:**

The e-Delphi method was chosen as it is an excellent technique for achieving consensus. The study began with an initial stage in which screening of a general practice clinical computer system and discussion groups with pharmacists from two ‘pharmacists in general practice’ sites identified 81 codes potentially relevant to general practice-based pharmacists’ work. Twenty-nine experts (pharmacists and pharmacy technicians from the two sites along with experts recruited through national committees) were then invited by e-mail to participate as a panel in three e-Delphi questionnaire rounds. In each round, panellists were asked to grade or rank codes and justify their choices. In every round, panellists were provided with anonymised feedback from the previous round which included their individual choices along with their co-panellists’ views. Final consensus (in Round 3) was defined as at least 80% agreement. Commentaries on the codes from all e-Delphi rounds were pooled together and analysed thematically.

**Results:**

Twenty-one individual panellists took part in the study (there were 12 responses in Round 1, 18 in Round 2 and 16 in Round 3). Commentaries on the codes included three themes: challenges and facilitators; level of detail; and activities related to funding. Consensus was achieved for ten codes, eight of which related to activities (general and disease specific medication reviews, monitoring of high-risk drugs and medicines reconciliation) and two to patient outcomes (presence of side effects and satisfactory understanding of medication).

**Conclusions:**

A formal consensus method revealed general practice-based pharmacists’ preferences for activity coding. Findings will inform policy so that any future shaping of activity coding for general practice-based pharmacists takes account of pharmacists’ actual needs and preferences.

**Electronic supplementary material:**

The online version of this article (10.1186/s12875-019-1008-6) contains supplementary material, which is available to authorized users.

## Background

In England, there is an ongoing drive to incorporate pharmacists into general practice (known as ‘family practice’ in some countries) which has been co-supported by the National Health Service (NHS) England, Health Education England, the Royal College of General Practitioners, the British Medical Association’s General Practitioners Committee and the Royal Pharmaceutical Society. In 2015, a national pilot scheme was introduced that partially covered the expenses of co-locating pharmacists into general practices as equal members in the multidisciplinary teams [1]. An amount of £31 million was invested in the pilot which formed a component of a wider plan [2] aiming to address needs in the primary care workforce (i.e. a shortage of roughly 8000 general practitioners (GPs) and, by 2040, an oversupply of 11,000 to 19,000 newly qualified pharmacists [3]). The pilot led to approximately 490 new general practice-based pharmacists’ posts across 90 sites which included approximately 658 general practices [4]. A pilot site, now ‘pharmacists in general practice’ site, includes a number of general practices participating in the pilot scheme as part of the same organisation. An example of a pilot site is a GP Federation (i.e. a group of practices, in the UK, working together within their geographical area as part of a collective entity). Following the pilot, the number of general practice-based pharmacists has increased as a result of a second roll-out phase [5]. The ultimate purpose with this second phase has been to integrate an additional 1500 pharmacists into general practices by 2020/21 thus having approximately one pharmacist per 30,000 patient population. The overall goal of the ‘pharmacists in general practice’ scheme is to reduce the workload of overburdened GPs (thus enabling them to manage their time-demands and focus where they are most needed, for example, diagnostics or treating patients with rare or very complex conditions) and ease patients’ access to healthcare services and checks [6]. Integrating pharmacists into general practice is also expanding to Wales, Scotland and Northern Ireland [7–10].

Historically, the pharmacy profession has been striving, across the globe, to gain recognition of pharmacists’ clinical roles by other healthcare professions and the public [11]. Every new clinical pharmacy service has therefore needed to demonstrate its effectiveness, efficiency and contribution to patient care to justify its necessity and continued funding [12, 13]. Internationally, the greatest challenges when integrating pharmacists into general practices have been overcoming GPs’ reluctance to accept pharmacists’ clinical interventions [14, 15] and patients’ unfamiliarity with pharmacists’ roles in this environment [16, 17]. To capture the impact of general practice-based pharmacists, and thus show their usefulness, a number of approaches have been employed internationally. In Australia, for example, the number of medication-related problems experienced by patients (such as incorrect medication or dose, adverse drug reactions and interactions) was measured before and six months after a consultation with a general practice-based pharmacist [18]. Measurements were done by interviewing patients and auditing their records. Results showed significant reductions in medication-related problems with pharmacists’ intervention. In Canada, a postal questionnaire was sent to GPs (at the 3^rd^, 12^th^ and 19^th^ month of pharmacists’ integration into general practices) asking them to grade their own and pharmacists’ contributions to a number of general practice-based activities [19]. Findings revealed the increasing contributions of pharmacists to diagnosis, prescribing, monitoring, medication reviews and education.

In the UK, NHS England has proposed a set of ten national Key Performance Indicators (KPIs) to evaluate the impact of the introduction of general practice-based pharmacists on patients, GPs and the wider healthcare system [20]. Eight of the KPIs are based on numerical components and two are survey-based (see Table 1). For the numerical KPIs, UK general practice-based pharmacists are required to record their day-to-day work on the clinical computer systems in the general practices by using pre-existing, non-pharmacy specific, electronic activity codes. SystmOne, EMIS and INPS Vision are the main clinical record systems used in UK general practices. At the time of writing, there has been no national agreement on what general practice-based pharmacists’ activities are worth capturing on a regular basis. A recent paper (2019) found that UK general practices still do not have a formal and/or common process for measuring the impact of their pharmacists (practices informally looked for reductions in their GPs’ workload or their improved performance in terms of quality inspections and national targets) [21]. A formal evaluation of the initial pilot scheme, based on the opinions of healthcare staff and patients, reported benefits for the practices (such as increased capacity, more focused workload for GPs and reductions in costs) and patients (such as availability of longer appointments with the same person in the practice) [22]. Although the evaluation looked at pharmacists’ perceptions about their roles, it made no quantitative measures of their activities. Moreover, a UK qualitative study revealed that the current coding options are not tailored to pharmacists’ work (available coding having pre-dated the ‘pharmacists in general practice’ scheme) and concluded that they are not fit for purpose to effectively identify the spectrum of pharmacists’ tasks within the general practice environment [23]. The purpose, therefore, of this study was to reach a broad consensus amongst experts on what general practice-based pharmacists’ activities should be recorded on the general practice clinical computer systems.

**Table 1:** National Key Performance Indicators (KPIs)

Numerical KPIs	
• Number of patient appointments with: General practitioner (GP), Practice Nurse, Clinical Pharmacist, Health Care Assistant/Advanced Nurse Practitioner	
• Impact on the percentage of patients who met the achievement indicator within the relevant Quality and Outcomes Framework-QOF (increase in the average QOF score)	
• Increase in total number of medication reviews	
• Decrease in the percentage of medication reviews undertaken by GPs	
• Increase in the total number of patients supported to develop care and support plans, including self-management	
• The rate of Accident & Emergency attendances per 1000 patients on GP register	
• Rate of emergency hospital admissions for selected long-term conditions as a proportion of patients per GP practice	
• Reduction in the number of patients attending ≥15 appointments with a GP over the previous two years by age group (0–9, 10–19, 20–39, 40–59, 60–69, 70–89, 90+)	
• Reduction in antibiotic prescribing rate (versus national rate per STARPU*)	
• Reduction in prescribing rate of anti-psychotic medications for patients with dementia or learning disabilities	
Survey-based KPIs	
• Patient satisfaction survey (patient experience)	
• GP survey (impact on workload, time, utilisation, job satisfaction)	

*STARPU (Specific Therapeutic Group Age-sex weightings Related Prescribing Units): a weighting system that takes into account the types of people receiving treatment within a specific therapeutic group to compare drug use between NHS organisations and practices

## Methods

### Study methodology

The Delphi method was selected for the current study because it enables consensus amongst experts (panellists) on a topic that lacks evidence [24–26]. The Delphi method involves an initial stage in which the recruited panel of experts identifies the spectrum of predominant problems which are then transformed into statements and ranked in a succession of consecutive questionnaire rounds. In each round, responses are influenced by controlled feedback from the previous round (i.e. panellists are offered an anonymised summary of their counterparts’ views). The study completes when a pre-defined agreement percentage is reached or after a pre-agreed number of rounds [27].

### Initial stage

Because there was no recent UK literature on pharmacists’ activities in general practice at the time of beginning this study, two members of the research team (GS-service lead and GDK-doctorate research student) screened the largest general practice computer system (SystmOne) and built up a list of 69 codes potentially relevant to general practice-based pharmacists’ work. The vast majority of codes were related to activities but there were also a few codes concerning patient outcomes that were included due to their potential high relevance to pharmacists’ activities in this setting as determined by GS. Face-to-face focus groups were then conducted with general practice-based pharmacists (from two West London sites) in which participants were asked to discuss which codes on the list might be useful and suggest any other pharmacists’ activities worth considering as coding options. These focus group discussions were audio-recorded, transcribed verbatim and analysed thematically (for detail, see reference [23]). A further 12 codes were generated from the focus group discussions. In total, a collection of 81 codes was assembled which made up the questionnaire for Round 1 of the Delphi study. An additional file presents all 81 codes (see Additional file 1). Each code formed a different item in the questionnaire. Two general practice-based pharmacists and one pharmacy technician pilot tested the questionnaire for Round 1 and any modifications made thereafter. All questionnaires were created using the platform of Online surveys (formerly known as Bristol Online Surveys).

### Expert panel

Clayton (1997) recommends 15 to 30 panellists as an ideal size for Delphi panels [28]. Twenty-nine people were identified as potential panel members for the current study, using the following criteria: pharmacists or pharmacy technicians working in general practice and involved in coding general practice-based pharmacists’ activities either at a local or national level. Invitees included all pharmacists and pharmacy technicians working across two West London sites (at the time approximately 17 eligible staff members) along with other national experts (12) holding senior general practice-based pharmacists’ roles and widely engaged on national committees. The West London sites were targeted for recruitment because both have working connections with the research team’s organisation (invitees from the West London sites included most of the focus group participants). The national experts were recruited through the Centre for Pharmacy Postgraduate Education and the Primary Care Pharmacy Association.

### Recruitment process

Participation was voluntary and all 29 experts were invited to participate in each round. All invitation e-mails for Round 1 were sent, on behalf of the research team, by the lead pharmacists in the two West London ‘pharmacists in general practice’ sites. The invitation included a direction to e-mail a member of the research team (GDK) if they wanted to be involved in the study. Once confirmatory e-mails had been received, the log-in details for access to the questionnaire were individually e-mailed (by GDK) directly to potential panellists.

In each subsequent round, GDK directly e-mailed the new log-in details of the updated questionnaires to those panellists involved in one of the previous round(s). Two weeks after the initial invitation, the lead pharmacists sent a follow-up invitation e-mail to the whole potential panel encouraging them to take part in the study.

### e-Delphi rounds

**Fig. 1:**
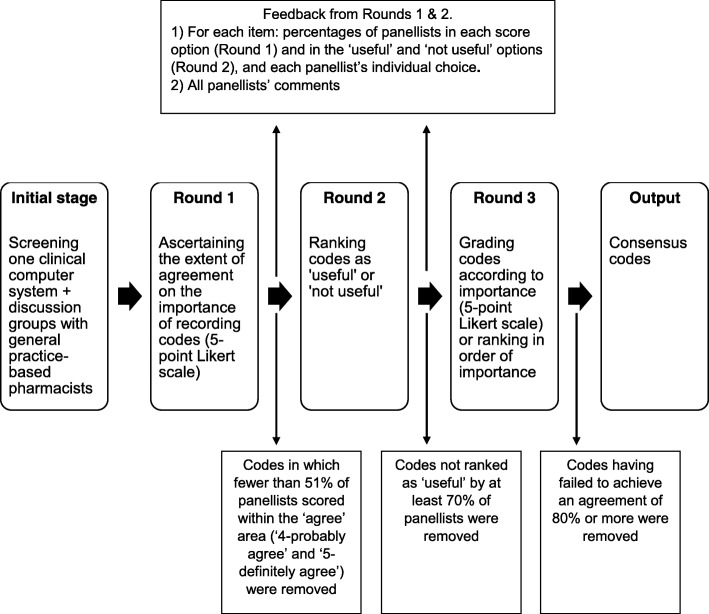
Process followed in the e-Delphi study of pharmacists’ activity in general practice

The study’s endpoint was consensus according to a preconceived criterion (agreement ≥80%). As literature reports that three Delphi iterations suffice for achieving consensus [29, 30], it was decided in advance to carry out a succession of three e-Delphi rounds in the current study. To foster the achievement of consensus, each round was different in terms of the questionnaire’s content and the threshold of agreement was progressively elevated (see Analysis of quantitative data below). In each round, panellists had the chance to explain their choice for each item and/or to provide general comments. Feedback from each previous round (see Fig. 1 for what it included) was organised into a PDF document and e-mailed, alongside log-in details, to panellists. Demographic data was collected in each round including overall years of practice as qualified healthcare professional, years of practice within the general practice environment, region of practice and roles/duties within general practice.

### Round 1

The questionnaire for this round asked panellists to report the extent to which they agreed that each of the proposed codes was important to record by using a 5-point Likert scale (1 = definitely disagree, 2 = probably disagree, 3 = neither agree nor disagree, 4 = probably agree, 5 = definitely agree). The questionnaire for this round can be found as an Additional file (see Additional file 2).

### Round 2

In this round, codes were grouped as per their context (e.g. codes relating to medication review, monitoring, patient outcomes etc.). Panellists were asked to characterise each code as ‘useful’ or ‘not useful’. The questionnaire for this round can be found as an Additional file (see Additional file 3).

### Round 3

In this round, codes were grouped similarly to Round 2 and panellists were asked to grade them according to their importance on a 5-point Likert scale (Very Important, Important, Moderately Important, Slightly Important, Not at all). For codes related to patient’s ability to manage medication, where duplication existed (i.e. multiple codes for the same meaning), panellists were instead asked to rank the available options in order of importance (1 = most important and 6 = least important). The questionnaire for this round can be found as an Additional file (see Additional file 4).

### Analysis of quantitative data

Descriptive statistics were employed to analyse quantitative data. In each round, the percentage of panellists in each score/ranking option was calculated automatically by the Online Surveys platform. The threshold of agreement was progressively elevated (51% in Round 1, 70% in Round 2 and 80% in Round 3). In detail, Round 1 codes in which fewer than 51% of panellists scored 4 (probably agree) and 5 (definitely agree) were removed. In Round 2, codes not characterised as ‘useful’ by at least 70% of panellists were removed. Final consensus was defined as at least 80% of the panellists in Round 3 scoring within the ‘importance’ area (i.e. ‘Very Important’ and ‘Important’). Similarly, for the ranking question in Round 3, consensus was implied if 80% or more of the panellists identified a certain option as belonging in the same position of the order of importance (i.e. at least 80% of panellists ranked an option as number 1, number 2 etc.).

Figure 1 summarises the process followed in this study, including the analysis process for the quantitative data.

### Analysis of qualitative data

Panellists in all Delphi rounds were given an identifier based on round, for example, Round 1, Panellist 1; Round 1, Panellist 2 etc. Panellists’ commentaries from all rounds were pooled together (on a hard copy) and analysed thematically. The six stages of thematic analysis as described by Braun and Clark were employed (familiarisation with data, coding, identifying themes, reviewing themes, defining themes and writing the report) [31]. In detail, commentaries were coded by GDK (the term ‘code’ or ‘coding’ in this context refers to the coding step of qualitative analysis [32] rather than to activity codes which formed the questionnaire items in each Delphi round). Codes were developed on the margins of the hard copies containing all commentaries and a single code was ascribed to every different meaning. Codes were confirmed by the whole research team before developing categories. All different codes were transferred to a Word® document and sorted into potential categories. Each category was highlighted with a different shading on the Word® document. Categories were eventually collapsed into themes with associated sub-themes. Themes were then refined and named collectively by the whole research team.

## Results

### Panellists

Responses were received from 21 individual panellists: 12 in Round 1, 18 in Round 2 and 16 in Round 3 (nine panellists followed through from Round 1 to 3). All panellists were employed in general practice and involved in capturing pharmacy services. Table 2 presents the demographics of the panellists, aggregated for reasons of anonymity.

**Table 2:** Demographics of the e-Delphi expert panel

	Years of practice as healthcare professional	Years of practice within the general practice environment	Region of practice
Pharmacists (19)	5 to 31 years	1 to 23 years	Essex, Greater Manchester, London, Midlands, South Wales
Pharmacy technicians (2)	> 10 years	< 5 years	London

Panellists reported a wide scope of practice, such as: various face-to-face consultation clinics including medication reviews, elements of diagnostics and performing regular home and care home visits, medication prescribing and monitoring duties including high risk drugs (e.g. immunosuppressants, lithium, warfarin) and overseeing the repeat prescription service; managing the discharge/clinical letter workflow and medicines reconciliations; education duties (e.g. training medical students or registrar GPs); consultancy work with healthcare professionals (e.g. medication queries) and leading multidisciplinary clinical meetings at a practice or broader level; audits and service reviews; telephone consultations with patients for advice on minor ailments and triage; and supporting the running of Patient Participation Groups. Pharmacy technicians, under the supervision of pharmacists, were involved in most of the above activities except for authorising prescriptions.

### Qualitative data

Commentaries were sorted into three main themes: challenges and facilitators; level of detail; and activities related to funding. These themes gave a better understanding of the reasons behind the selection of certain codes as the most important options and allowed extrapolation to relevant recommendations.

### Challenges and facilitators

The panellists discussed several factors that might act as challenges or facilitators in the process of recording activities.

With regards to challenges, there was a fear that activity coding might complicate a pharmacist’s working day (i.e. an extra daily task) and generate additional time-requirements.

*The use of codes is time consuming. For me, coding would add complexity to [my] working day.* (Round 1, Panellist 1)

Some panellists claimed that the greater the available options (e.g. codes differentiating activities to a larger degree), the more laborious the process of coding would become.

*Coding should not be too onerous as it gets difficult to maintain that high level of reporting.* (Round 2, Panellist 9)

It was also reported that entering a code might occasionally act as a distractor from focusing on the patient during consultations.

*Having codes to use may mean concentrating to complete templates rather than actually delving into patients’ needs and care plans.* (Round 1, Panellist 12)

Memorising codes was another reported challenge.

*Hard to see how all these codes will be used in the GP practice setting as pharmacists would have to remember them.* (Round 1, Panellist 5)

There were concerns that codes are likely to be used irregularly amongst different pharmacists and/or practices hence complicating any subsequent data collection.

*The data extracted might be limited due to an irregular use of codes.* (Round 1, Panellist 1)

Panellists also mentioned that some codes referred to tasks not frequently carried out by general practice-based pharmacists. Examples included synchronising repeat medications, believed to mainly be a task for administrators, and reviewing community pharmacists’ Medicine Use Reviews (MURs), infrequently undertaken by general practice-based pharmacists. A MUR is a service offered by community pharmacists in the UK and involves adherence-focused reviews with patients on certain medicine groups.

Concerning facilitators, it was suggested that a national activity coding template would ease the process of coding. All options would be in the same place and therefore easily accessible.

*It would be extremely useful to develop a national template with all codes on for easy access.* (Round 1, Panellist 4)

There was, however, a fear that a national template might not entirely account for local needs that individual practices and/or pharmacists might experience.

*Is there such a thing as one size fits all with regards to a template or should we create an a la carte menu for people to pick from?* (Round 1, Panellist 12)

The need to create clear definitions for all codes was also highlighted.

*All codes need to be clearly defined.* (Round 1, Panellist 3)

### Level of detail

The amount of detail the codes should include was frequently commented upon and conflicting opinions were present.

For example, there was discussion about medication review codes and whether or not these should:

- Be more specifically attributed to the person carrying out the review (e.g. pharmacist, GP).
  *It’s useful to know as quick glance at the code whether GP or pharmacist did the medication review.* (Round 3, Panellist 7)
  *The details of the clinician on a code are only relevant for auditing. For everyday practice, the system will identify the user as the type of clinician.* (Round 2, Panellist 11)
- Define the exact disease area (e.g. asthma, depression etc.) for which the review was conducted.
  *Some disease-specific medication review codes are helpful especially if pharmacists are working in the earlier part of their employment as independent prescribers and sticking to their scope of practice.* (Round 2, Panellist 9)
  *A general medication review code encompasses all conditions. If wanted to, you can see what condition-medication you are reviewing from notes (and can be searched on the electronic systems) without need to code specifically.* (Round 2, Panellist 8)
- Indicate the level of the review (i.e. presence or absence of the patient during a review).
  *Information is limited without the patient [being] present [at a medication review], so it’s good to code the patient’s presence or absence.* (Round 3, Panellist 8)
  *Reviewing medication even without the patient adds some value so segregating it [patient’s presence] out of the [general] medication review code is of limited value.* (Round 2, Panellist 9)

### Activities related to funding

Panellists emphasised the importance of primarily coding activities associated with the availability of funding streams for general practices. They provided characteristic examples of funding-related activities such as the monitoring of high-risk drugs which was viewed as a part of commissioned ‘out-of-hospital services’ (i.e. healthcare services offered by UK non-hospital providers, such as general practices, that attract NHS funding). In addition, medication reviews for patients with certain conditions, such as asthma and diabetes, were believed to qualify for Quality and Outcomes Framework (QOF) funding. QOF is a program for English, Welsh and Northern Irish general practices that incentivises clinical excellence.

*We have a huge array of things to do daily and much of it relates to practice funding so have to be secure in doing this. This [e-Delphi] study needs to search against codes already in use for the purposes of QOF/‘out-of-hospital services’ etc. to get more accurate data.* (Round 1, Panellist 1)

*Codes already used in practice for purposes of getting funding are useful.* (Round 3, Panellist 4)

### Activity codes

Of the 81 codes in Round 1, 59 codes made it through to Round 2 (58 from Round 1 and one added following panellists’ comments) and 34 codes made it through to Round 3 (33 from Round 2 and one added following panellists’ comments). Additional file 1 presents percentage agreement for each code in all rounds. Final consensus (in Round 3) was reached on ten codes (see Table 3). Table 4 presents the Round 3 codes that failed to achieve final consensus. In addition, there was no clear hierarchy in ranking for the importance of any of the following Round 3 codes which were subsequently discarded: ‘able to manage medication’; ‘drug compliance good’; ‘unable to manage medication’; ‘difficulty managing medication’; ‘uses medication administration system’; and ‘needs assistance with medication regimen adherence’.

**Table 3:** Codes for which final consensus (agreement ≥80%) was reached

• Medication review done	
• Medication review done by pharmacist	
• Asthma medication review	
• Chronic Obstructive Pulmonary Disease (COPD) medication review	
• Diabetes medication review	
• Depression medication review	
• High-risk drug monitoring	
• Medicines reconciliation post-discharge with notes	
• Has shown side effects from medication	
• Patient understands why taking all medication

**Table 4:** Codes in Round 3 that failed to achieve final consensus (categorised by percentage agreement*)

Agreement 70–80%	• Antipsychotic medication review • Polypharmacy medication review • Medication changed • New medication added • Medicines reconciliation performed
Agreement 60–70%	• Medicines adherence checked • Advice about drug treatment • Advice about side effects of drug treatment • Medication review without patient • Anticoagulation medication review • Drug changed to cost effective alternative • Medication stopped • Medication stopped-side effect • Medication error
Agreement 50–60%	• No drug side effect reported • Synchronisation of repeat medication • Contact with the local community pharmacy • Medicine Use Review (MUR) done by community pharmacist

*Percentage agreement indicates how many panellists identified a code as ‘Very Important’ and ‘Important’

## Discussion

Of the ten codes for which consensus was reached, eight relate to activities and two to patient outcomes. The selected patient outcome codes refer to the presence of side effects and to the satisfactory understanding of medications. Panellists did not provide reasons for why they viewed these two patient outcome codes as important, however, this might be because these codes are seen as standard checks for a pharmacist to ensure the patient’s adherence to medication.

The eight chosen activity codes refer to only three distinct activities: medication review, monitoring of high-risk drugs and medicines reconciliation. In contrast, general practice-based pharmacists across the Dudley Clinical Commissioning Group (i.e. a UK clinically-led body, part of the NHS, in charge of designing and commissioning healthcare services for the local area) were found to code at least 20 different activities ranging from direct patient care tasks to duties related to education, quality assurance, repeat prescribing and waste management [33]. Activity codes in Dudley, however, were determined exclusively by the service lead without taking account of any validation by pharmacists or any external expert input, which our study has done.

The activities favoured for coding are mainly funding-related tasks: medication reviews (especially for conditions viewed by panellists as the top priorities in QOF: asthma, COPD, diabetes and depression) along with the monitoring of high-risk drugs which was viewed by panellists as a priority in ‘out-of-hospital services’. The fact that one of the national KPIs accounts for the ability to meet QOF targets might have arguably influenced panellists’ choice of codes. These results support the finding that there is increasing engagement of UK general practice-based pharmacists with incentive programs related to funding acquisition for their employer practices [34]. Panellists, however, provided no comments on why they chose medicines reconciliation (not viewed as funding-related) as an activity to code.

As shown in Additional file 1, the majority of codes that made it through to Round 3 had also good percentage agreement in Rounds 2 and 1. Some codes, however, with high percentage agreement in Round 2 did not get final consensus in Round 3. For instance, the ‘no drug side effect reported’ code did not maintain high percentage agreement in contrast to its opposite ‘has shown side effects from medication’ code. This could have been because it is more important for pharmacists to record the presence rather than the absence of side effects, for example, to alert the rest of the general practice-based team. Codes describing the patient’s ability to manage medications were also discarded in Round 3, perhaps because they were not deemed as direct measures of a pharmacist’s activity (these codes describe patient behaviours). It is also worth noting that panellists finally selected a medicines reconciliation code pointing out the availability of patient notes rather than the generic ‘medicines reconciliation performed’. This choice makes sense, terminology-wise, because medicines reconciliation cannot properly be done without access to patient notes [35]. Panellists rejected codes describing medication reviews for anticoagulants and antipsychotics, despite the fact that one of the KPIs requires general practice-based pharmacists to reduce antipsychotic prescribing. These codes were possibly excluded because patients on antipsychotics or anticoagulants would be under hospital or specialist care.

General practice-based pharmacists were against having to deal with a large number of codes because they would be onerous, provide more detail than necessary and be less likely to have universal uptake. A few, higher-order codes were preferred instead. For instance, panellists did not select codes describing specific actions taken during a medication review (such as altering medication, ascertaining adherence and offering advice about treatment), most likely because these could be covered and implied under the higher-order ‘medication review done’ code. Probably for the same reason of avoiding large numbers of codes, panellists excluded codes indicating the level of a review and codes believed to describe rare tasks for general practice-based pharmacists such as synchronising repeat medications and reviewing community pharmacists’ MURs. Although communication with community pharmacists was recognised during the initial focus groups as an important element of general practice-based pharmacist’s role [23], panellists did not consider it important enough to code how often it happens. The ‘contact with local community pharmacy’ code was rejected potentially because interactions between general practice-based and community pharmacists are extremely frequent [34] and using codes would have made coding quite time consuming.

To dispel fears about the negative impact that the use of codes could have on day-to-day workflow, additional simplification of the activity coding process could be beneficial. For example, an Australian public hospital employed barcode technology to facilitate capture of pharmacists’ activities [36]. Technology can ease reference to codes and accelerate their entry into clinical computer systems thus making activity coding a smoother process for general practice-based pharmacists.

### Implications

This study has shown consensus on a number of activity (and patient outcome) codes. Clear definitions of codes along with policies on their use need to be created (e.g. explanations of terminology, instances or prerequisites for entering each code) to encourage an unvarying application of codes and hence facilitate any subsequent data collection.

### Strengths and limitations

This is the first study that has followed an acknowledged consensus method to determine general practice-based pharmacists’ preferences concerning activity coding. As the Delphi method requires, the panel used in the current study included some of the key experts on the topic who have been following the evolution of UK general practice-based pharmacists’ roles for many years. Consequently, findings reflect real needs/requirements concerning capturing pharmacists’ impact in general practice and, additionally, account for diverse levels of experience (people relatively new in general practice were also represented in the study) as well as different geographical areas of practice. The study explored the views of the whole pharmacy team in general practice including pharmacy technicians who are increasingly contributing to general practice-based activities [37].

As it was an entirely UK-based study, findings might not be generalisable to other countries due to possible differences between healthcare systems. Individual elements, however, will still be useful wherever attempts are being made to implement and justify general practice-based pharmacists’ services. For example, aspects of the findings might be useful to Australia, Canada and New Zealand which all have formal programs for integrating and testing pharmacists’ services in general practice [38–40]. The original list of activity codes was mainly based on only one clinical computer system and there might be additional codes present on other systems. Panellists, however, had the chance throughout the Delphi study (and in the initial focus groups before the actual Delphi rounds) to suggest any other activities of importance to capture. Therefore, it is anticipated that the study has identified the important activities for recording general practice-based pharmacists’ impact regardless of the clinical computer system used.

## Conclusions

This study followed a formal consensus technique to offer insight into needs and preferences of general practice-based pharmacists with regards to activity coding. Final consensus was reached for ten codes with a notable preference for codes required for obtaining general practice funding. These findings will be useful for general practice-based pharmacists wanting to align their activity coding practices with options widely recognised as useful. These findings will also inform policy that attempts to shape activity coding for general practice-based pharmacists by considering pharmacists’ actual needs and preferences.

## Additional files

Additional file 1: Codes in all rounds of the e-Delphi study and percentage agreement on each one. Description of data: This additional file consists of a table that presents all codes that were present in the e-Delphi’s rounds, including percentage agreement that each code received in each round. (DOCX 17 kb)

Additional file 2: Round 1 questionnaire. Description of data: This additional file consists of the questionnaire used for Round 1 of the e-Delphi study. (PDF 294 kb)

Additional file 3: Round 2 questionnaire. Description of data: This additional file consists of the questionnaire used for Round 2 of the e-Delphi study. (PDF 104 kb)

Additional file 4: Round 3 questionnaire. Description of data: This additional file consists of the questionnaire used for Round 3 of the e-Delphi study. (PDF 99 kb)

## Abbreviations

COPD: Chronic Obstructive Pulmonary Disease
GP: General Practitioner
KPI: Key Performance Indicator
MUR: Medicine Use Review
NHS: National Health Service
QOF: Quality and Outcomes Framework
STARPU: Specific Therapeutic Group Age-sex weightings Related Prescribing Units
