# Supplementary material for: Capturing pharmacists’ impact in general practice: an e-Delphi study to attempt to reach consensus amongst experts about what activities to record
Source: BMC Fam Pract. 2019 Sep 9;20:126. doi: 10.1186/s12875-019-1008-6 (PMC6734337; doi:10.1186/s12875-019-1008-6)
Supplement: Supplementary file 1 — Codes in all rounds of the e-Delphi study and percentage agreement on each one. Description of data: This additional file consists of a table that presents all codes that were present in the e-Delphi’s rounds, including percentage agreement that each code received in each round. (DOCX 17 kb) [file 12875_2019_1008_MOESM1_ESM.docx]

**Additional file 1: Codes in all rounds of the e-Delphi study and percentage agreement on each one**

| **Codes** | **Round 1 (%*)** | **Round 2 (%**)** | **Round 3 (%***)** |
| --- | --- | --- | --- |
| Drug side effect-acceptable to patient | 42 |  |  |
| On four or more medications | 16 |  |  |
| Medication satisfactory | 8 |  |  |
| Needs assistance with medication concordance | 50 |  |  |
| Patient medication advice | 42 |  |  |
| Medication review done by medicines management pharmacist | 41 |  |  |
| Medication review with patient | 42 |  |  |
| Respiratory disease medication review | 42 |  |  |
| Diabetic medicine | 25 |  |  |
| Hypertension six month review | 50 |  |  |
| Bisphosphonate medication review | 50 |  |  |
| Osteoporosis medication review | 50 |  |  |
| Other medication review | 17 |  |  |
| Efficacy of all medication checked | 25 |  |  |
| Repeat medication check | 50 |  |  |
| Answer to GP medication-related query^#^ | 50 |  |  |
| Hypertension monitoring check done | 50 |  |  |
| Adjustment of a patient’s medication inside the framework of drug monitoring^#^ | 34 |  |  |
| Medication on discharge letter | 50 |  |  |
| Medication optimisation | 50 |  |  |
| Medication management plan in situ | 34 |  |  |
| Clinical check on a patient^#^ | 16 |  |  |
| Drug therapy discontinued | 50 |  |  |
| Able to use medication | 72 | 58.8 |  |
| Advice to continue with drug treatment | 67 | 61.1 |  |
| Medication discussed with pharmacist | 84 | 66.7 |  |
| Advice to GP to change patient medication | 75 | 61.1 |  |
| Medication counselling | 84 | 55.6 |  |
| Medication review done by pharmacy technician | 67 | 50 |  |
| Medication review of medical notes | 67 | 55.6 |  |
| Cardiac medication review | 58 | 58.8 |  |
| Coronary heart disease medication review | 58 | 52.9 |  |
| Epilepsy medication review | 66 | 61.1 |  |
| Dementia medication review | 75 | 66.7 |  |
| Drug compliance checked | 54 | 61.1 |  |
| Indication for each drug checked | 66 | 50 |  |
| Medication increased | 59 | 61.1 |  |
| Medication decreased | 67 | 61.1 |  |
| Repeat prescription reviewed by pharmacist | 58 | 55.6 |  |
| Medicine list reviewed for inefficient use/unwanted medicines | 59 | 50 |  |
| Cost alternative medication switch | 83 | 38.9 |  |
| Medication monitoring | 84 | 55.6 |  |
| Any other kind of drug monitoring^#^ | 67 | 11.1 |  |
| Blood pressure monitoring | 67 | 44.4 |  |
| Medicines reconciliation post-discharge with patient^#^ | 83 | 61.1 |  |
| Medicines reconciliation on admission to a nursing home^#^ | 75 | 50 |  |
| Stop an unnecessary request for an antibiotic^#^ | 66 | 50 |  |
| Review of a MUR sent by the community pharmacy to the clinical pharmacist in the general practice^#^ | 84 | 64.7 |  |
| Seen by pharmacist | 67 | 61.1 |  |
| Able to manage medication | 83 | 83.3 | 31.3 |
| Unable to manage medication | 75 | 83.3 | 40 |
| Difficulty managing medication | 83 | 72.2 | 46.7 |
| Uses medication administration system | 75 | 77.8 | 40 |
| Drug compliance good | 58 | 77.8 | 33.4 |
| Needs assistance with medication regimen adherence | 75 | 88.9 | 13.4 |
| Patient understands why taking all medication | 100 | 100 | 81.3 |
| No drug side effect reported | 83 | 83.3 | 56.3 |
| Has shown side effects from medication | 91 | 77.8 | 87.5 |
| Advice about side effects of drug treatment | 83 | 88.9 | 68.8 |
| Advice about drug treatment | 75 | 72.2 | 62.5 |
| Medication review done | 66 | 77.8 | 87.5 |
| Medication review done by pharmacist | 91 | 77.8 | 81.3 |
| Medication review without patient | 75 | 77.8 | 62.5 |
| Asthma medication review | 67 | 77.8 | 80 |
| COPD medication review^#^ | 67 | 77.8 | 80 |
| Anticoagulation medication review | 67 | 72.2 | 66.7 |
| Diabetes medication review | 66 | 77.8 | 80 |
| Antipsychotic medication review | 75 | 72.2 | 73.3 |
| Depression medication review | 75 | 72.2 | 80 |
| Polypharmacy medication review | 67 | 72.2 | 75.1 |
| Medicines adherence checked | 83 | 83.3 | 62.5 |
| Medication changed | 66 | 77.8 | 75.1 |
| New medication added | 59 | 72.2 | 75 |
| Medication stopped-side effect | 83 | 83.3 | 68.3 |
| Medication stopped |  |  | 68.8 |
| Synchronisation of repeat medication | 92 | 88.9 | 56.3 |
| Drug changed to cost effective alternative |  | 77.8 | 62.6 |
| High-risk drug monitoring^#^ | 100 | 94.4 | 93.8 |
| Medicines reconciliation performed | 92 | 83.3 | 75.1 |
| Medicines reconciliation post-discharge with notes^#^ | 83 | 72.2 | 87.5 |
| Medication error | 92 | 72.2 | 68.8 |
| Contact with the local community pharmacy^#^ | 75 | 72.2 | 50 |
| Medicine Use Review (MUR) done by community pharmacist | 84 | 72.2 | 50.1 |
|  | **Number of panellists in Round 1: 12** | **Number of panellists in Round 2: 18** | **Number of panellists in Round 3: 16** |

*Threshold: agreement ≥ 51%

**Threshold: agreement ≥ 70%

***Consensus: agreement ≥ 80%

^#^Code generated during face-to-face, focus group discussions
