# Supplementary material for: Capturing pharmacists’ impact in general practice: an e-Delphi study to attempt to reach consensus amongst experts about what activities to record
Source: BMC Fam Pract. 2019 Sep 9;20:126. doi: 10.1186/s12875-019-1008-6 (PMC6734337; doi:10.1186/s12875-019-1008-6)
Supplement: Supplementary file 2 — Round 1 questionnaire. Description of data: This additional file consists of the questionnaire used for Round 1 of the e-Delphi study. (PDF 294 kb) [file 12875_2019_1008_MOESM2_ESM.pdf]

What pharmacists' activities (codes) should be recorded? Working towards investigating pharmacist input into the general practice environment - an e-Delphi study (Round 1)

Page 1: Welcome

Dear Sir/Madam

Welcome to this questionnaire about pharmacists' activities in general practice. We want to know which ones you think are the most important to record. This will then allow the measurement of the impact of pharmacists working in the general practice environment.

The questionnaire is built from existing activity/outcome codes from a variety of sources which means that, at this stage, there is a lot of repetition and overlap. The aim, with your help, is to reduce the number of coded activities/outcomes and identify a few that will be essential for accurately capturing your impact in general practice without recording being burdensome.

This is the first round of a Delphi study, which is anticipated to run over 2-3 rounds. Consensus is defined as getting 80% or greater agreement on an activity/outcome to be recorded.

The questionnaire can be saved part way through (by clicking on the "Finish later" option which can be found at the bottom of each page) and returned to later. It will take approximately 10-15 minutes to complete. The questionnaire is part of the research for my PhD in Pharmacy at the University of Reading.

Many thanks in advance for your time.

Yours sincerely,

George Karampatakis

Chief Investigator - PhD student

Reading School of Pharmacy

Supervisors: Prof. Kath Ryan, Dr. Nilesh Patel

Page 2: Data protection

Please note that data collected in this questionnaire will be stored only on the secure, password protected Bristol Online Survey (BOS) platform.

The completed online questionnaires will be accessible only to me (George Karampatakis), and, after being separated from the “token”, to my supervisors (Kath Ryan, Nilesh Patel).

Data analysis and information made public or included in research outputs will use aggregated results. In any dissemination of the survey data, all identifying information from individual responses to this survey will be removed. Great care will be taken to either aggregate or coarsely categorize potential identifying information, e.g. participants’ years of experience will only be reported as a range. No other sensitive information will be collected.

Cookies and personal data stored by your Web browser are not used in this survey.

Page 3: How to complete this questionnaire

This questionnaire has two parts. The main part focusses on the activities/outcomes to be recorded (page 5). The final part (page 7) asks for some brief professional information about you.

In the main part we ask your opinion using rating scales. Please select the grade that best represents your opinion. Please read each question carefully and answer all the questions to the best of your ability. For each question you will also have the opportunity to write a short comment, if you wish, about why you gave a specific grade. For any general comments there is a "General comments" section right after the main questionnaire (page 6).

There are no right or wrong responses. We are just interested in your personal point of view and would really appreciate your honest views so that the data are robust. You can now proceed to the main questionnaire section.

Page 4: Token

1. Please enter your "token" (e-mailed to you by George Karampatakis) ✱ *Required*

Page 5: Questions

To what extent do you agree that the following codes (questions 2-82) are important to record?

2. Able to use medication

Please don't select more than 1 answer(s) per row.

	1 (definitely disagree)	2 (probably disagree)	3 (neither agree nor disagree)	4 (probably agree)	5 (definitely agree)
Score	☐	☐	☐	☐	☐

2.a. Brief explanation of your score (optional)

3. Able to manage medication

Please don't select more than 1 answer(s) per row.

	1 (definitely disagree)	2 (probably disagree)	3 (neither agree nor disagree)	4 (probably agree)	5 (definitely agree)
Score	☐	☐	☐	☐	☐

3.a. Brief explanation of your score (optional)

4. Unable to manage medication

Please don't select more than 1 answer(s) per row.

	1 (definitely disagree)	2 (probably disagree)	3 (neither agree nor disagree)	4 (probably agree)	5 (definitely agree)
Score	☐	☐	☐	☐	☐

4.a. Brief explanation of your score (optional)

5. Difficulty managing medication

Please don't select more than 1 answer(s) per row.

	1 (definitely disagree)	2 (probably disagree)	3 (neither agree nor disagree)	4 (probably agree)	5 (definitely agree)
Score	☐	☐	☐	☐	☐

5.a. Brief explanation of your score (optional)

6. Uses medication administration system (e.g. dosette box)

Please don't select more than 1 answer(s) per row.

	1 (definitely disagree)	2 (probably disagree)	3 (neither agree nor disagree)	4 (probably agree)	5 (definitely agree)
Score	☐	☐	☐	☐	☐

6.a. Brief explanation of your score (optional)

7. Patient understands why taking all medication

Please don't select more than 1 answer(s) per row.

	1 (definitely disagree)	2 (probably disagree)	3 (neither agree nor disagree)	4 (probably agree)	5 (definitely agree)
Score	☐	☐	☐	☐	☐

7.a. Brief explanation of your score (optional)

8. No drug side effect reported

Please don't select more than 1 answer(s) per row.

	1 (definitely disagree)	2 (probably disagree)	3 (neither agree nor disagree)	4 (probably agree)	5 (definitely agree)
Score	☐	☐	☐	☐	☐

8.a. Brief explanation of your score (optional)

9. Has shown side effects from medication

Please don't select more than 1 answer(s) per row.

	1 (definitely disagree)	2 (probably disagree)	3 (neither agree nor disagree)	4 (probably agree)	5 (definitely agree)
Score	☐	☐	☐	☐	☐

9.a. Brief explanation of your score (optional)

10. Drug side effect - acceptable to patient

Please don't select more than 1 answer(s) per row.

	1 (definitely disagree)	2 (probably disagree)	3 (neither agree nor disagree)	4 (probably agree)	5 (definitely agree)
Score	☐	☐	☐	☐	☐

10.a. Brief explanation of your score (optional)

11. Advice about side effects of drug treatment

Please don't select more than 1 answer(s) per row.

	1 (definitely disagree)	2 (probably disagree)	3 (neither agree nor disagree)	4 (probably agree)	5 (definitely agree)
Score	☐	☐	☐	☐	☐

11.a. Brief explanation of your score (optional)

12. On four or more medications

Please don't select more than 1 answer(s) per row.

	1 (definitely disagree)	2 (probably disagree)	3 (neither agree nor disagree)	4 (probably agree)	5 (definitely agree)
Score	☐	☐	☐	☐	☐

12.a. Brief explanation of your score (optional)

13. Medication satisfactory

Please don't select more than 1 answer(s) per row.

	1 (definitely disagree)	2 (probably disagree)	3 (neither agree nor disagree)	4 (probably agree)	5 (definitely agree)
Score	☐	☐	☐	☐	☐

13.a. Brief explanation of your score (optional)

14. Drug compliance checked

Please don't select more than 1 answer(s) per row.

	1 (definitely disagree)	2 (probably disagree)	3 (neither agree nor disagree)	4 (probably agree)	5 (definitely agree)
Score	☐	☐	☐	☐	☐

14.a. Brief explanation of your score (optional)

15. Medicines adherence checked

Please don't select more than 1 answer(s) per row.

	1 (definitely disagree)	2 (probably disagree)	3 (neither agree nor disagree)	4 (probably agree)	5 (definitely agree)
Score	☐	☐	☐	☐	☐

15.a. Brief explanation of your score (optional)

16. Drug compliance good

Please don't select more than 1 answer(s) per row.

	1 (definitely disagree)	2 (probably disagree)	3 (neither agree nor disagree)	4 (probably agree)	5 (definitely agree)
Score	☐	☐	☐	☐	☐

16.a. Brief explanation of your score (optional)

17. Needs assistance with medication regimen adherence

Please don't select more than 1 answer(s) per row.

	1 (definitely disagree)	2 (probably disagree)	3 (neither agree nor disagree)	4 (probably agree)	5 (definitely agree)
Score	☐	☐	☐	☐	☐

17.a. Brief explanation of your score (optional)

18. Needs assistance with medication concordance

Please don't select more than 1 answer(s) per row.

	1 (definitely disagree)	2 (probably disagree)	3 (neither agree nor disagree)	4 (probably agree)	5 (definitely agree)
Score	☐	☐	☐	☐	☐

18.a. Brief explanation of your score (optional)

19. Advice about drug treatment

Please don't select more than 1 answer(s) per row.

	1 (definitely disagree)	2 (probably disagree)	3 (neither agree nor disagree)	4 (probably agree)	5 (definitely agree)
Score	☐	☐	☐	☐	☐

19.a. Brief explanation of your score (optional)

20. Patient medication advice

Please don't select more than 1 answer(s) per row.

	1 (definitely disagree)	2 (probably disagree)	3 (neither agree nor disagree)	4 (probably agree)	5 (definitely agree)
Score	☐	☐	☐	☐	☐

20.a. Brief explanation of your score (optional)

21. Advice to continue with drug treatment

Please don't select more than 1 answer(s) per row.

	1 (definitely disagree)	2 (probably disagree)	3 (neither agree nor disagree)	4 (probably agree)	5 (definitely agree)
Score	☐	☐	☐	☐	☐

21.a. Brief explanation of your score (optional)

22. Medication discussed with pharmacist

Please don't select more than 1 answer(s) per row.

	1 (definitely disagree)	2 (probably disagree)	3 (neither agree nor disagree)	4 (probably agree)	5 (definitely agree)
Score	☐	☐	☐	☐	☐

22.a. Brief explanation of your score (optional)

23. Seen by pharmacist

Please don't select more than 1 answer(s) per row.

	1 (definitely disagree)	2 (probably disagree)	3 (neither agree nor disagree)	4 (probably agree)	5 (definitely agree)
Score	☐	☐	☐	☐	☐

23.a. Brief explanation of your score (optional)

24. Medication review done

Please don't select more than 1 answer(s) per row.

	1 (definitely disagree)	2 (probably disagree)	3 (neither agree nor disagree)	4 (probably agree)	5 (definitely agree)
Score	☐	☐	☐	☐	☐

24.a. Brief explanation of your score (optional)

25. Medication review done by pharmacist

Please don't select more than 1 answer(s) per row.

	1 (definitely disagree)	2 (probably disagree)	3 (neither agree nor disagree)	4 (probably agree)	5 (definitely agree)
Score	☐	☐	☐	☐	☐

25.a. Brief explanation of your score (optional)

26. Medication review done by pharmacy technician

Please don't select more than 1 answer(s) per row.

	1 (definitely disagree)	2 (probably disagree)	3 (neither agree nor disagree)	4 (probably agree)	5 (definitely agree)
Score	☐	☐	☐	☐	☐

26.a. Brief explanation of your score (optional)

27. Medication review done by medicines management pharmacist

Please don't select more than 1 answer(s) per row.

	1 (definitely disagree)	2 (probably disagree)	3 (neither agree nor disagree)	4 (probably agree)	5 (definitely agree)
Score	☐	☐	☐	☐	☐

27.a. Brief explanation of your score (optional)

28. Medication review with patient

Please don't select more than 1 answer(s) per row.

	1 (definitely disagree)	2 (probably disagree)	3 (neither agree nor disagree)	4 (probably agree)	5 (definitely agree)
Score	☐	☐	☐	☐	☐

28.a. Brief explanation of your score (optional)

29. Medication review without patient

Please don't select more than 1 answer(s) per row.

	1 (definitely disagree)	2 (probably disagree)	3 (neither agree nor disagree)	4 (probably agree)	5 (definitely agree)
Score	☐	☐	☐	☐	☐

29.a. Brief explanation of your score (optional)

30. Medication review of medical notes

Please don't select more than 1 answer(s) per row.

	1 (definitely disagree)	2 (probably disagree)	3 (neither agree nor disagree)	4 (probably agree)	5 (definitely agree)
Score	☐	☐	☐	☐	☐

30.a. Brief explanation of your score (optional)

31. Respiratory disease medication review

Please don't select more than 1 answer(s) per row.

	1 (definitely disagree)	2 (probably disagree)	3 (neither agree nor disagree)	4 (probably agree)	5 (definitely agree)
Score	☐	☐	☐	☐	☐

31.a. Brief explanation of your score (optional)

32. Asthma medication review

Please don't select more than 1 answer(s) per row.

	1 (definitely disagree)	2 (probably disagree)	3 (neither agree nor disagree)	4 (probably agree)	5 (definitely agree)
Score	☐	☐	☐	☐	☐

32.a. Brief explanation of your score (optional)

33. COPD medication review

Please don't select more than 1 answer(s) per row.

	1 (definitely disagree)	2 (probably disagree)	3 (neither agree nor disagree)	4 (probably agree)	5 (definitely agree)
Score	☐	☐	☐	☐	☐

33.a. Brief explanation of your score (optional)

34. Cardiac medication review

Please don't select more than 1 answer(s) per row.

	1 (definitely disagree)	2 (probably disagree)	3 (neither agree nor disagree)	4 (probably agree)	5 (definitely agree)
Score	☐	☐	☐	☐	☐

34.a. Brief explanation of your score (optional)

35. Coronary heart disease medication review

Please don't select more than 1 answer(s) per row.

	1 (definitely disagree)	2 (probably disagree)	3 (neither agree nor disagree)	4 (probably agree)	5 (definitely agree)
Score	☐	☐	☐	☐	☐

35.a. Brief explanation of your score (optional)

36. Anticoagulant medication review

Please don't select more than 1 answer(s) per row.

	1 (definitely disagree)	2 (probably disagree)	3 (neither agree nor disagree)	4 (probably agree)	5 (definitely agree)
Score	☐	☐	☐	☐	☐

36.a. Brief explanation of your score (optional)

37. Diabetes medication review

Please don't select more than 1 answer(s) per row.

	1 (definitely disagree)	2 (probably disagree)	3 (neither agree nor disagree)	4 (probably agree)	5 (definitely agree)
Score	☐	☐	☐	☐	☐

37.a. Brief explanation of your score (optional)

38. Diabetic medicine

Please don't select more than 1 answer(s) per row.

	1 (definitely disagree)	2 (probably disagree)	3 (neither agree nor disagree)	4 (probably agree)	5 (definitely agree)
Score	☐	☐	☐	☐	☐

38.a. Brief explanation of your score (optional)

39. Hypertension six month review

Please don't select more than 1 answer(s) per row.

	1 (definitely disagree)	2 (probably disagree)	3 (neither agree nor disagree)	4 (probably agree)	5 (definitely agree)
Score	☐	☐	☐	☐	☐

39.a. Brief explanation of your score (optional)

40. Antipsychotic medication review

Please don't select more than 1 answer(s) per row.

	1 (definitely disagree)	2 (probably disagree)	3 (neither agree nor disagree)	4 (probably agree)	5 (definitely agree)
Score	☐	☐	☐	☐	☐

40.a. Brief explanation of your score (optional)

41. Depression medication review

Please don't select more than 1 answer(s) per row.

	1 (definitely disagree)	2 (probably disagree)	3 (neither agree nor disagree)	4 (probably agree)	5 (definitely agree)
Score	☐	☐	☐	☐	☐

41.a. Brief explanation of your score (optional)

42. Epilepsy medication review

Please don't select more than 1 answer(s) per row.

	1 (definitely disagree)	2 (probably disagree)	3 (neither agree nor disagree)	4 (probably agree)	5 (definitely agree)
Score	☐	☐	☐	☐	☐

42.a. Brief explanation of your score (optional)

43. Dementia medication review

Please don't select more than 1 answer(s) per row.

	1 (definitely disagree)	2 (probably disagree)	3 (neither agree nor disagree)	4 (probably agree)	5 (definitely agree)
Score	☐	☐	☐	☐	☐

43.a. Brief explanation of your score (optional)

44. Bisphosphonate medication review

Please don't select more than 1 answer(s) per row.

	1 (definitely disagree)	2 (probably disagree)	3 (neither agree nor disagree)	4 (probably agree)	5 (definitely agree)
Score	☐	☐	☐	☐	☐

44.a. Brief explanation of your score (optional)

45. Osteoporosis medication compliance review

Please don't select more than 1 answer(s) per row.

	1 (definitely disagree)	2 (probably disagree)	3 (neither agree nor disagree)	4 (probably agree)	5 (definitely agree)
Score	☐	☐	☐	☐	☐

45.a. Brief explanation of your score (optional)

46. Polypharmacy medication review

Please don't select more than 1 answer(s) per row.

	1 (definitely disagree)	2 (probably disagree)	3 (neither agree nor disagree)	4 (probably agree)	5 (definitely agree)
Score	☐	☐	☐	☐	☐

46.a. Brief explanation of your score (optional)

47. Other medication review

Please don't select more than 1 answer(s) per row.

	1 (definitely disagree)	2 (probably disagree)	3 (neither agree nor disagree)	4 (probably agree)	5 (definitely agree)
Score	☐	☐	☐	☐	☐

47.a. Brief explanation of your score (optional)

48. Medicine list reviewed for inefficient use/unwanted medicines

Please don't select more than 1 answer(s) per row.

	1 (definitely disagree)	2 (probably disagree)	3 (neither agree nor disagree)	4 (probably agree)	5 (definitely agree)
Score	☐	☐	☐	☐	☐

48.a. Brief explanation of your score (optional)

49. Efficacy of all medication checked

Please don't select more than 1 answer(s) per row.

	1 (definitely disagree)	2 (probably disagree)	3 (neither agree nor disagree)	4 (probably agree)	5 (definitely agree)
Score	☐	☐	☐	☐	☐

49.a. Brief explanation of your score (optional)

50. Indication for each drug checked

Please don't select more than 1 answer(s) per row.

	1 (definitely disagree)	2 (probably disagree)	3 (neither agree nor disagree)	4 (probably agree)	5 (definitely agree)
Score	☐	☐	☐	☐	☐

50.a. Brief explanation of your score (optional)

51. Repeat medication check

Please don't select more than 1 answer(s) per row.

	1 (definitely disagree)	2 (probably disagree)	3 (neither agree nor disagree)	4 (probably agree)	5 (definitely agree)
Score	☐	☐	☐	☐	☐

51.a. Brief explanation of your score (optional)

52. Repeat prescription reviewed by pharmacist

Please don't select more than 1 answer(s) per row.

	1 (definitely disagree)	2 (probably disagree)	3 (neither agree nor disagree)	4 (probably agree)	5 (definitely agree)
Score	☐	☐	☐	☐	☐

52.a. Brief explanation of your score (optional)

53. Synchronisation of repeat medication

Please don't select more than 1 answer(s) per row.

	1 (definitely disagree)	2 (probably disagree)	3 (neither agree nor disagree)	4 (probably agree)	5 (definitely agree)
Score	☐	☐	☐	☐	☐

53.a. Brief explanation of your score (optional)

54. Medication error

Please don't select more than 1 answer(s) per row.

	1 (definitely disagree)	2 (probably disagree)	3 (neither agree nor disagree)	4 (probably agree)	5 (definitely agree)
Score	☐	☐	☐	☐	☐

54.a. Brief explanation of your score (optional)

55. Advice to GP to change patient medication

Please don't select more than 1 answer(s) per row.

	1 (definitely disagree)	2 (probably disagree)	3 (neither agree nor disagree)	4 (probably agree)	5 (definitely agree)
Score	☐	☐	☐	☐	☐

55.a. Brief explanation of your score (optional)

56. Answer to GP medication-related query

Please don't select more than 1 answer(s) per row.

	1 (definitely disagree)	2 (probably disagree)	3 (neither agree nor disagree)	4 (probably agree)	5 (definitely agree)
Score	☐	☐	☐	☐	☐

56.a. Brief explanation of your score (optional)

57. Medication counselling

Please don't select more than 1 answer(s) per row.

	1 (definitely disagree)	2 (probably disagree)	3 (neither agree nor disagree)	4 (probably agree)	5 (definitely agree)
Score	☐	☐	☐	☐	☐

57.a. Brief explanation of your score (optional)

58. Medication monitoring

Please don't select more than 1 answer(s) per row.

	1 (definitely disagree)	2 (probably disagree)	3 (neither agree nor disagree)	4 (probably agree)	5 (definitely agree)
Score	☐	☐	☐	☐	☐

58.a. Brief explanation of your score (optional)

59. High-risk drug monitoring performed (e.g. blood levels of lithium, phenytoin)

Please don't select more than 1 answer(s) per row.

	1 (definitely disagree)	2 (probably disagree)	3 (neither agree nor disagree)	4 (probably agree)	5 (definitely agree)
Score	☐	☐	☐	☐	☐

59.a. Brief explanation of your score (optional)

60. Any other kind of drug monitoring performed (e.g. blood pressure for ACE-inhibitors, liver function tests for statins)

Please don't select more than 1 answer(s) per row.

	1 (definitely disagree)	2 (probably disagree)	3 (neither agree nor disagree)	4 (probably agree)	5 (definitely agree)
Score	☐	☐	☐	☐	☐

60.a. Brief explanation of your score (optional)

61. Blood pressure monitoring

Please don't select more than 1 answer(s) per row.

	1 (definitely disagree)	2 (probably disagree)	3 (neither agree nor disagree)	4 (probably agree)	5 (definitely agree)
Score	☐	☐	☐	☐	☐

61.a. Brief explanation of your score (optional)

62. Hypertension monitoring check done

Please don't select more than 1 answer(s) per row.

	1 (definitely disagree)	2 (probably disagree)	3 (neither agree nor disagree)	4 (probably agree)	5 (definitely agree)
Score	☐	☐	☐	☐	☐

62.a. Brief explanation of your score (optional)

63. Adjustment of a patient's medication inside the framework of drug monitoring

Please don't select more than 1 answer(s) per row.

	1 (definitely disagree)	2 (probably disagree)	3 (neither agree nor disagree)	4 (probably agree)	5 (definitely agree)
Score	☐	☐	☐	☐	☐

63.a. Brief explanation of your score (optional)

64. Medicines reconciliation performed

Please don't select more than 1 answer(s) per row.

	1 (definitely disagree)	2 (probably disagree)	3 (neither agree nor disagree)	4 (probably agree)	5 (definitely agree)
Score	☐	☐	☐	☐	☐

64.a. Brief explanation of your score (optional)

65. Medicines reconciliation post-discharge with patient

Please don't select more than 1 answer(s) per row.

	1 (definitely disagree)	2 (probably disagree)	3 (neither agree nor disagree)	4 (probably agree)	5 (definitely agree)
Score	☐	☐	☐	☐	☐

65.a. Brief explanation of your score (optional)

66. Medicines reconciliation post-discharge with notes

Please don't select more than 1 answer(s) per row.

	1 (definitely disagree)	2 (probably disagree)	3 (neither agree nor disagree)	4 (probably agree)	5 (definitely agree)
Score	☐	☐	☐	☐	☐

66.a. Brief explanation of your score (optional)

67. Medication on discharge letter

Please don't select more than 1 answer(s) per row.

	1 (definitely disagree)	2 (probably disagree)	3 (neither agree nor disagree)	4 (probably agree)	5 (definitely agree)
Score	☐	☐	☐	☐	☐

67.a. Brief explanation of your score (optional)

68. Medicines reconciliation on admission to a nursing home

Please don't select more than 1 answer(s) per row.

	1 (definitely disagree)	2 (probably disagree)	3 (neither agree nor disagree)	4 (probably agree)	5 (definitely agree)
Score	☐	☐	☐	☐	☐

68.a. Brief explanation of your score (optional)

69. Medication changed

Please don't select more than 1 answer(s) per row.

	1 (definitely disagree)	2 (probably disagree)	3 (neither agree nor disagree)	4 (probably agree)	5 (definitely agree)
Score	☐	☐	☐	☐	☐

69.a. Brief explanation of your score (optional)

70. Cost alternative medication switch

Please don't select more than 1 answer(s) per row.

	1 (definitely disagree)	2 (probably disagree)	3 (neither agree nor disagree)	4 (probably agree)	5 (definitely agree)
Score	☐	☐	☐	☐	☐

70.a. Brief explanation of your score (optional)

71. New medication added

Please don't select more than 1 answer(s) per row.

	1 (definitely disagree)	2 (probably disagree)	3 (neither agree nor disagree)	4 (probably agree)	5 (definitely agree)
Score	☐	☐	☐	☐	☐

71.a. Brief explanation of your score (optional)

72. Medication increased

Please don't select more than 1 answer(s) per row.

	1 (definitely disagree)	2 (probably disagree)	3 (neither agree nor disagree)	4 (probably agree)	5 (definitely agree)
Score	☐	☐	☐	☐	☐

72.a. Brief explanation of your score (optional)

73. Medication decreased

Please don't select more than 1 answer(s) per row.

	1 (definitely disagree)	2 (probably disagree)	3 (neither agree nor disagree)	4 (probably agree)	5 (definitely agree)
Score	☐	☐	☐	☐	☐

73.a. Brief explanation of your score (optional)

74. Medication stopped - side effect

Please don't select more than 1 answer(s) per row.

	1 (definitely disagree)	2 (probably disagree)	3 (neither agree nor disagree)	4 (probably agree)	5 (definitely agree)
Score	☐	☐	☐	☐	☐

74.a. Brief explanation of your score (optional)

75. Drug therapy discontinued

Please don't select more than 1 answer(s) per row.

	1 (definitely disagree)	2 (probably disagree)	3 (neither agree nor disagree)	4 (probably agree)	5 (definitely agree)
Score	☐	☐	☐	☐	☐

75.a. Brief explanation of your score (optional)

76. Stop an unnecessary request for an antibiotic (e.g. for rescue packs)

Please don't select more than 1 answer(s) per row.

	1 (definitely disagree)	2 (probably disagree)	3 (neither agree nor disagree)	4 (probably agree)	5 (definitely agree)
Score	☐	☐	☐	☐	☐

76.a. Brief explanation of your score (optional)

77. Medication optimisation

Please don't select more than 1 answer(s) per row.

	1 (definitely disagree)	2 (probably disagree)	3 (neither agree nor disagree)	4 (probably agree)	5 (definitely agree)
Score	☐	☐	☐	☐	☐

77.a. Brief explanation of your score (optional)

78. Medication management plan in situ

Please don't select more than 1 answer(s) per row.

	1 (definitely disagree)	2 (probably disagree)	3 (neither agree nor disagree)	4 (probably agree)	5 (definitely agree)
Score	☐	☐	☐	☐	☐

78.a. Brief explanation of your score (optional)

79. Clinical check on a patient

Please don't select more than 1 answer(s) per row.

	1 (definitely disagree)	2 (probably disagree)	3 (neither agree nor disagree)	4 (probably agree)	5 (definitely agree)
Score	☐	☐	☐	☐	☐

79.a. Brief explanation of your score (optional)

80. Contact with the local community pharmacy

Please don't select more than 1 answer(s) per row.

	1 (definitely disagree)	2 (probably disagree)	3 (neither agree nor disagree)	4 (probably agree)	5 (definitely agree)
Score	☐	☐	☐	☐	☐

80.a. Brief explanation of your score (optional)

81. Medicine use review (MUR) done by community pharmacist

Please don't select more than 1 answer(s) per row.

	1 (definitely disagree)	2 (probably disagree)	3 (neither agree nor disagree)	4 (probably agree)	5 (definitely agree)
Score	☐	☐	☐	☐	☐

81.a. Brief explanation of your score (optional)

82. Review of a MUR sent by the community pharmacy to the clinical pharmacist in the general practice

Please don't select more than 1 answer(s) per row.

	1 (definitely disagree)	2 (probably disagree)	3 (neither agree nor disagree)	4 (probably agree)	5 (definitely agree)
Score	☐	☐	☐	☐	☐

82.a. Brief explanation of your score (optional)

Page 6: General comments

83. Please provide any general comments/ideas/thoughts you have

Page 7: Demographics

84. Please state your overall years of practice as a health professional ✱ *Required*

85. Please state your years of practice within the general practice environment ✱ *Required*

86. Please state your current role(s) within the general practice environment ✱ *Required*

87. Please state the region of England where you practise ✱ *Required*

Page 8: Thank you

Dear Sir/Madam

I would like to sincerely thank you for completing the first round of the Delphi study.

Your participation will significantly contribute to demonstrating pharmacy input within general practices.

Once all the questionnaires have been collected and analysed, I will be contacting you again for the second round of the study.

In the meantime and in case you have any further questions/concerns, please do not hesitate to contact me (G.D.Karampatakis@pgr.reading.ac.uk)

Again many thanks for your time.

Yours sincerely,

George Karampatakis

Chief Investigator - PhD student

Reading School of Pharmacy

Supervisors: Prof. Kath Ryan, Dr. Nilesh Patel
