# Supplementary material for: Capturing pharmacists’ impact in general practice: an e-Delphi study to attempt to reach consensus amongst experts about what activities to record
Source: BMC Fam Pract. 2019 Sep 9;20:126. doi: 10.1186/s12875-019-1008-6 (PMC6734337; doi:10.1186/s12875-019-1008-6)
Supplement: Supplementary file 3 — Round 2 questionnaire. Description of data: This additional file consists of the questionnaire used for Round 2 of the e-Delphi study. (PDF 104 kb) [file 12875_2019_1008_MOESM3_ESM.pdf]

# What pharmacists' activities (codes) should be recorded? Working towards investigating pharmacist input into the general practice environment - an e-Delphi study (Round 2)

## Page 1: Welcome

Dear Sir/Madam

Welcome to the second round of this e-Delphi study. With your help, our aim is to further reduce the number of coded activities or patient outcomes and identify those codes thought essential for capturing your impact in general practice. You can still take part in this round irrespective of whether you completed the first e-Delphi round.

The questionnaire can be saved part way through (by clicking on the "Finish later" option which can be found at the bottom of each page) and returned to later. The questionnaire will take approximately 5-10 minutes to complete.

Many thanks in advance for your time.

Yours sincerely

George Karampatakis

Reading School of Pharmacy

Supervisors: Prof. Kath Ryan, Dr. Nilesh Patel

# Page 2: Data protection

Please note that data collected in this questionnaire will be stored only on the secure, password protected Bristol Online Survey (BOS) platform.

The completed online questionnaires will be accessible only to me (George Karampatakis), and, after being separated from the “token”, to my supervisors (Kath Ryan, Nilesh Patel).

Data analysis and information made public or included in research outputs will use aggregated results. In any dissemination of the survey data, all identifying information from individual responses to this survey will be removed. Great care will be taken to either aggregate or coarsely categorize potential identifying information, e.g. participants’ years of experience will only be reported as a range. No other sensitive information will be collected.

Cookies and personal data stored by your Web browser are not used in this survey.

# Page 3: How to complete this questionnaire

This questionnaire has two parts. The main part focuses on the activities/outcomes to be recorded (page 5-13). The final part (page 15) asks for some brief professional information about you.

In the main part we ask you to rank certain codes as "useful" or "not useful". Please read each question carefully and answer all the questions to the best of your ability. For each question you will also have the opportunity to write a short comment, if you wish, about your choice(s). For any general comments there is a "General comments" section right after the main questionnaire (page 14).

There are no right or wrong responses. We are just interested in your personal point of view and would really appreciate your honest views so that the data is robust. You can now proceed to the main questionnaire section.

# Page 4: Token

1. Please enter your "token" (e-mailed to you by George Karampatakis) ✱ *Required*

# Page 5: Patient outcome codes

*2.* Please state whether each of the following options (related to patient's ability to understand and manage medications) is useful or not

Please don't select more than 1 answer(s) per row.

| | Useful | Not useful |
|---|---|---|
| Able to use medication | ☐ | ☐ |
| Able to manage medication | ☐ | ☐ |
| Unable to manage medication | ☐ | ☐ |
| Difficulty managing medication | ☐ | ☐ |
| Uses medication administration system | ☐ | ☐ |
| Drug compliance good | ☐ | ☐ |
| Needs assistance with medication regimen adherence | ☐ | ☐ |
| Patient understands why taking all medication | ☐ | ☐ |

*2.a.* Brief explanation of your choice(s) *Optional*

*3.* Please state whether each of the following options (related to side effect outcomes) is useful or not

Please don't select more than 1 answer(s) per row.

| | Useful | Not useful |
|---|---|---|
| No drug side effect reported | ☐ | ☐ |
| Has shown side effects from medication | ☐ | ☐ |

3.a. Brief explanation of your choice(s) *Optional*

# Page 6: Medication advice codes

*4.* Please state whether each of the following options (related to advice given) is useful or not

Please don't select more than 1 answer(s) per row.

| | Useful | Not useful |
|---|---|---|
| Advice about side effects of drug treatment | ☐ | ☐ |
| Advice about drug treatment | ☐ | ☐ |
| Advice to continue with drug treatment | ☐ | ☐ |
| Medication discussed with pharmacist | ☐ | ☐ |
| Advice to GP to change patient medication | ☐ | ☐ |
| Medication counselling | ☐ | ☐ |

*4.a.* Brief explanation of your choice(s) *Optional*

# Page 7: Medication review codes

*5.* Please state whether each of the following options (related to who has conducted the review) is useful or not

Please don't select more than 1 answer(s) per row.

| | Useful | Not useful |
|---|---|---|
| Medication review done | ☐ | ☐ |
| Mediation review done by pharmacist | ☐ | ☐ |
| Medication review done by pharmacy technician | ☐ | ☐ |

*5.a.* Brief explanation of your choice(s) *Optional*

*6.* Please state whether each of the following options (related to the level of the review) is useful or not

Please don't select more than 1 answer(s) per row.

| | Useful | Not useful |
|---|---|---|
| Medication review without patient | ☐ | ☐ |
| Medication review of medical notes | ☐ | ☐ |

*6.a.* Brief explanation of your choice(s) *Optional*

7. Please state whether each of the following options (related to specific conditions) is useful or not

Please don't select more than 1 answer(s) per row.

| | Useful | Not useful |
|---|---|---|
| Asthma medication review | ☐ | ☐ |
| COPD medication review | ☐ | ☐ |
| Cardiac medication review | ☐ | ☐ |
| Coronary heart disease medication review | ☐ | ☐ |
| Anticoagulation medication review | ☐ | ☐ |
| Diabetes medication review | ☐ | ☐ |
| Antipsychotic medication review | ☐ | ☐ |
| Depression medication review | ☐ | ☐ |
| Epilepsy medication review | ☐ | ☐ |
| Dementia medication review | ☐ | ☐ |
| Polypharmacy medication review | ☐ | ☐ |

7.a. Brief explanation of your choice(s) *Optional*

8. Please state whether each of the following options (related to adherence ascertainment) is useful or not

Please don't select more than 1 answer(s) per row.

| | Useful | Not useful |
|---|---|---|
| Drug compliance checked | ☐ | ☐ |
| Medicines adherence checked | ☐ | ☐ |

*8.a.* Brief explanation of your choice(s) *Optional*

*9.* Please state whether each of the following options (related to other potential activities during medication reviews) is useful or not

Please don't select more than 1 answer(s) per row.

| | Useful | Not useful |
|---|---|---|
| Indication for each drug checked | ☐ | ☐ |
| Medication changed | ☐ | ☐ |
| New medication added | ☐ | ☐ |
| Medication increased | ☐ | ☐ |
| Medication decreased | ☐ | ☐ |
| Medication stopped - side effect | ☐ | ☐ |

*9.a.* Brief explanation of your choice(s) *Optional*

10. Please state whether each of the following options (related to repeat medications) is useful or not

Please don't select more than 1 answer(s) per row.

| | Useful | Not useful |
|---|---|---|
| Repeat prescription reviewed by pharmacist | ☐ | ☐ |
| Synchronisation of repeat medication | ☐ | ☐ |

10.a. Brief explanation of your choice(s) *Optional*

11. Please state whether each of the following options (related to medicinal waste/costs) is useful or not

Please don't select more than 1 answer(s) per row.

| | Useful | Not useful |
|---|---|---|
| Medicine list reviewed for inefficient use/unwanted medicines | ☐ | ☐ |
| Cost alternative medication switch | ☐ | ☐ |
| Drug changed to cost effective alternative | ☐ | ☐ |

11.a. Brief explanation of your choice(s) *Optional*

# Page 8: Monitoring codes

*12.* Please state whether each of the following options is useful or not

Please don't select more than 1 answer(s) per row.

| | Useful | Not useful |
|---|---|---|
| Medication monitoring | ☐ | ☐ |
| High-risk drug monitoring | ☐ | ☐ |
| Any other kind of drug monitoring | ☐ | ☐ |
| Blood pressure monitoring | ☐ | ☐ |

*12.a.* Brief explanation of your choice(s) *Optional*

# Page 9: Medicine reconciliation codes

13. Please state whether each of the following options is useful or not

Please don't select more than 1 answer(s) per row.

| | Useful | Not useful |
|---|---|---|
| Medicines reconciliation performed | ☐ | ☐ |
| Medicines reconciliation post-discharge with patient | ☐ | ☐ |
| Medicines reconciliation post-discharge with notes | ☐ | ☐ |
| Medicines reconciliation on admission to a nursing home | ☐ | ☐ |

13.a. Brief explanation of your choice(s) *Optional*

# Page 10: Code related to medication errors

*14.* Please state whether the following code is useful or not

Please don't select more than 1 answer(s) per row.

| | Useful | Not useful |
|---|---|---|
| Medication error | ☐ | ☐ |

*14.a.* Brief explanation of your choice *Optional*

# Page 11: Code related to antibiotics

*15.* Please state whether the following code is useful or not

Please don't select more than 1 answer(s) per row.

| | Useful | Not useful |
|---|---|---|
| Stop an unnecessary request for an antibiotic | ☐ | ☐ |

*15.a.* Brief explanation of your choice *Optional*

# Page 12: Codes related to interactions between practice-based and community pharmacists

16. Please state whether each of the following options is useful or not

Please don't select more than 1 answer(s) per row.

| | Useful | Not useful |
|---|---|---|
| Contact with the local community pharmacy | ☐ | ☐ |
| Medicine use review (MUR) done by community pharmacist | ☐ | ☐ |
| Review of a MUR sent by the community pharmacy to the clinical pharmacist in the general practice | ☐ | ☐ |

16.a. Brief explanation of your choice(s) *Optional*

# Page 13: Code related to contact with a practice-based pharmacist

*17.* Please state whether the following code is useful or not

Please don't select more than 1 answer(s) per row.

| | Useful | Not useful |
|---|---|---|
| Seen by pharmacist | ☐ | ☐ |

*17.a.* Brief explanation of your choice *Optional*

# Page 14: General comments

18. Please provide any general comments/ideas/thoughts you have *Optional*

## Page 15: Demographics

19. Please state your overall years of practice as a healthcare professional * *Required*

20. Please state your years of practice within the general practice environment * *Required*

21. Please state the region of England where you practise * *Required*

22. Please state your current role(s) within the general practice environment * *Required*

23. Please provide some examples of activities that you carry out in general practice on a regular basis ✱ *Required*

# Page 16: Thank you

Dear Sir/Madam

I would like to sincerely thank you for completing the second round of the Delphi study.

Your participation will significantly contribute to demonstrating pharmacy input within general practices.

Once all the questionnaires have been collected and analysed, I will be contacting you again for the third (and last) round of the study.

In the meantime, and in case you have any further questions/concerns, please do not hesitate to contact me (G.D.Karampatakis@pgr.reading.ac.uk)

Again, many thanks for your time.

Yours sincerely,

George Karampatakis

Reading School of Pharmacy

Supervisors: Prof. Kath Ryan, Dr. Nilesh Patel

---
