# Supplementary material for: Capturing pharmacists’ impact in general practice: an e-Delphi study to attempt to reach consensus amongst experts about what activities to record
Source: BMC Fam Pract. 2019 Sep 9;20:126. doi: 10.1186/s12875-019-1008-6 (PMC6734337; doi:10.1186/s12875-019-1008-6)
Supplement: Supplementary file 4 — Round 3 questionnaire. Description of data: This additional file consists of the questionnaire used for Round 3 of the e-Delphi study. (PDF 99 kb) [file 12875_2019_1008_MOESM4_ESM.pdf]

What pharmacists' activities (codes) should be recorded? Working towards investigating pharmacist input into the general practice environment - an e-Delphi study (Round 3)

Page 1: Welcome

Dear Sir/Madam

Welcome to the third (and FINAL) round of this e-Delphi study. With your help, our aim is to identify a few codes that will not duplicate each over and that will effectively capture your impact in general practice (without recording being burdensome).

This is a much shorter questionnaire (as compared to the previous rounds). It will take approximately 3 - 5 minutes to complete.

You can still take part in this round irrespective of whether you completed the previous e-Delphi round(s).

The questionnaire can be saved part way through (by clicking on the "Finish later" option which can be found at the bottom of each page) and returned to later.

Many thanks in advance for your time.

Yours sincerely

George Karampatakis

Reading School of Pharmacy

Supervisors: Prof. Kath Ryan, Dr. Nilesh Patel

Page 2: Data protection

Please note that data collected in this questionnaire will be stored only on the secure, password protected Bristol Online Survey (BOS) platform.

The completed online questionnaires will be accessible only to me (George Karampatakis), and, after being separated from the “token”, to my supervisors (Kath Ryan, Nilesh Patel).

Data analysis and information made public or included in research outputs will use aggregated results. In any dissemination of the survey data, all identifying information from individual responses to this survey will be removed. No other sensitive information will be collected.

Cookies and personal data stored by your Web browser are not used in this survey.

Page 3: How to complete this questionnaire

Before completing the questionnaire, it is very IMPORTANT that you read the feedback from the previous round (i.e. overall percentages and comments provided by participants) that has been e-mailed to you by George Karampatakis.

In the main part of the questionnaire (pages 5 - 13), we seek your opinion on 34 codes. All questions will ask you to grade codes according to their importance (except for one in which you will be asked to rank the options in order of importance). Please read each question carefully and answer all the questions to the best of your ability (especially those in which duplication exists - PLEASE identify those codes that BEST illustrate the proposed activity or outcome).

For each question you will also have the opportunity to write a short comment about your choice(s). For any general comments there is a "General comments" section right after the main questionnaire (page 14).

Please note that there are no right or wrong responses.

You can now proceed to the main questionnaire section.

Page 4: Token

1. Please enter your "token" (e-mailed to you by George Karampatakis) ✱
Required

Page 5: Codes related to patient's adherence and ability to manage medication

2. Please grade the following code according to its importance

Please don't select more than 1 answer(s) per row.

	Very Important	Important	Moderately Important	Slightly Important	Not at all
Medicines adherence checked	☐	☐	☐	☐	☐

2.a. Brief explanation of your choice *Optional*

3. Please rank the following codes (patient outcomes) in order of importance, where 1 is most important and 6 is least important

Please don't select more than 1 answer(s) per row.

Please don't select more than 1 answer(s) in any single column.

	1	2	3	4	5	6
Able to manage medication	☐	☐	☐	☐	☐	☐
Drug compliance good	☐	☐	☐	☐	☐	☐
Unable to manage medication	☐	☐	☐	☐	☐	☐

Difficulty managing medication	☐	☐	☐	☐	☐	☐
Uses medication administration system	☐	☐	☐	☐	☐	☐
Needs assistance with medication regimen adherence	☐	☐	☐	☐	☐	☐

3.a. Brief explanation of your choice(s) *Optional*

Page 6: Codes related to side effect outcomes

4. Please grade the following codes according to their importance (N.B. consider if both are equally important or not)

Please don't select more than 1 answer(s) per row.

	Very Important	Important	Moderately Important	Slightly Important	Not at all
No drug side effect reported	☐	☐	☐	☐	☐
Has shown side effects from medication	☐	☐	☐	☐	☐

4.a. Brief explanation of your choice(s) *Optional*

Page 7: Code related to patient's understanding of medication

5. Please grade the following code according to its importance

Please don't select more than 1 answer(s) per row.

	Very Important	Important	Moderately Important	Slightly Important	Not at all
Patient understands why taking all medication	☐	☐	☐	☐	☐

5.a. Brief explanation of your choice *Optional*

Page 8: Codes related to advice given

6. Please grade the following codes according to their importance (N.B. consider whether or not the first option encompasses everything)

Please don't select more than 1 answer(s) per row.

	Very Important	Important	Moderately Important	Slightly Important	Not at all
Advice about drug treatment	☐	☐	☐	☐	☐
Advice about side effects of drug treatment	☐	☐	☐	☐	☐

6.a. Brief explanation of your choice(s) *Optional*

Page 9: Medication review codes

7. Please grade the following code (related to the level of the review) according to its importance

Please don't select more than 1 answer(s) per row.

	Very Important	Important	Moderately Important	Slightly Important	Not at all
Medication review without patient	☐	☐	☐	☐	☐

7.a. Brief explanation of your choice *Optional*

8. Please grade the following codes according to their importance (N.B. online systems can identify the type of clinician)

Please don't select more than 1 answer(s) per row.

	Very Important	Important	Moderately Important	Slightly Important	Not at all
Medication review done	☐	☐	☐	☐	☐
Medication review done by pharmacist	☐	☐	☐	☐	☐

8.a. Brief explanation of your choice(s) *Optional*

9. Please grade the following codes according to their importance (N.B. consider whether or not this level of detail is necessary)

Please don't select more than 1 answer(s) per row.

	Very Important	Important	Moderately Important	Slightly Important	Not at all
Asthma medication review	☐	☐	☐	☐	☐
COPD medication review	☐	☐	☐	☐	☐
Anticoagulation medication review	☐	☐	☐	☐	☐
Diabetes medication review	☐	☐	☐	☐	☐
Antipsychotic medication review	☐	☐	☐	☐	☐
Depression medication review	☐	☐	☐	☐	☐
Polypharmacy medication review	☐	☐	☐	☐	☐

9.a. Brief explanation of your choice(s) *Optional*

10. Please grade the following codes (related to other potential activities during medication reviews) according to their importance

Please don't select more than 1 answer(s) per row.

	Very Important	Important	Moderately Important	Slightly Important	Not at all
Medication changed	☐	☐	☐	☐	☐
New Medication added	☐	☐	☐	☐	☐
Synchronisation of repeat medication	☐	☐	☐	☐	☐
Drug changed to cost effective alternative	☐	☐	☐	☐	☐

10.a. Brief explanation of your choice(s) *Optional*

11. Please grade the following codes (related to stopping medications) according to their importance (N.B. consider whether or not the first option encompasses everything)

Please don't select more than 1 answer(s) per row.

	Very Important	Important	Moderately Important	Slightly Important	Not at all
Medication stopped	☐	☐	☐	☐	☐
Medication stopped - side effect	☐	☐	☐	☐	☐

11.a. Brief explanation of your choice(s) *Optional*

Page 10: Monitoring code

12. Please grade the following code according to its importance

Please don't select more than 1 answer(s) per row.

	Very Important	Important	Moderately Important	Slightly Important	Not at all
High-risk drug monitoring	☐	☐	☐	☐	☐

12.a. Brief explanation of your choice *Optional*

Page 11: Medicine reconciliation codes

13. Please grade the following codes according to their importance (N.B. consider whether or not the first option encompasses everything)

Please don't select more than 1 answer(s) per row.

	Very Important	Important	Moderately Important	Slightly Important	Not at all
Medicines reconciliation performed	☐	☐	☐	☐	☐
Medicines reconciliation post-discharge with notes	☐	☐	☐	☐	☐

13.a. Brief explanation of your choice(s) *Optional*

Page 12: Code related to medication errors

14. Please grade the following code according to its importance

Please don't select more than 1 answer(s) per row.

	Very Important	Important	Moderately Important	Slightly Important	Not at all
Medication error	☐	☐	☐	☐	☐

14.a. Brief explanation of your choice *Optional*

Page 13: Codes related to interactions between practice-based and community pharmacists

15. Please grade the following codes according to their importance

Please don't select more than 1 answer(s) per row.

	Very Important	Important	Moderately Important	Slightly Important	Not at all
Contact with the local community pharmacy	☐	☐	☐	☐	☐
Medicine Use Review (MUR) done by community pharmacist	☐	☐	☐	☐	☐

15.a. Brief explanation of your choice(s) *Optional*

Page 14: General comments

16. Please provide any general comments/ideas/thoughts you have *Optional*

Page 15: Demographics

17. Please state the region of the UK where you currently practise ✱ *Required*

Page 16: Thank you

Dear Sir/Madam

I would like to sincerely thank you for completing the final round of the Delphi study.

Once all data is properly analysed, you will receive an e-mail containing a brief summary of the findings.

In the meantime, and in case you have any further questions/concerns, please do not hesitate to contact me (G.D.Karampatakis@pgr.reading.ac.uk)

Again, many thanks for your time.

Yours sincerely,

George Karampatakis

Reading School of Pharmacy

Supervisors: Prof. Kath Ryan, Dr. Nilesh Patel
